# Efficacy of personalized rTMS to enhance upper limb function in subacute stroke patients: a protocol for a multi-center, randomized controlled study

**DOI:** 10.3389/fneur.2024.1427142

**Published:** 2024-07-03

**Authors:** Ho Seok Lee, Dae Hyun Kim, Han Gil Seo, Sun Im, Yeun Jie Yoo, Na Young Kim, Jungsoo Lee, Donghyeon Kim, Hae-Yeon Park, Mi-Jeong Yoon, Young Seok Kim, Hyunjin Kim, Won Hyuk Chang

**Affiliations:** ^1^Department of Physical and Rehabilitation Medicine, Center for Prevention and Rehabilitation, Heart Vascular Stroke Institute, Samsung Medical Center, Sungkyunkwan University School of Medicine, Seoul, Republic of Korea; ^2^Department of Rehabilitation Medicine, Seoul National University College of Medicine, Seoul National University Hospital, Seoul, Republic of Korea; ^3^Department of Rehabilitation Medicine, Bucheon St. Mary’s Hospital, College of Medicine, The Catholic University of Korea, Seoul, Republic of Korea; ^4^Department of Rehabilitation Medicine, St. Vincent’s Hospital, College of Medicine, The Catholic University of Korea, Seoul, Republic of Korea; ^5^Department of Rehabilitation Medicine, Yongin Severance Hospital, Yonsei University College of Medicine, Yongin, Republic of Korea; ^6^Department of Medical IT Convergence Engineering, Kumoh National Institute of Technology, Gumi, Republic of Korea; ^7^NEUROPHET Inc., Research Institute, Seoul, Republic of Korea; ^8^Department of Health Science and Technology, Department of Medical Device Management and Research, SAIHST, Sungkyunkwan University, Seoul, Republic of Korea

**Keywords:** stroke, rTMS, functional reserve, neurorehabiliation, personalized medicine

## Abstract

**Background:**

Repetitive transcranial magnetic stimulation (rTMS) is widely used therapy to enhance motor deficit in stroke patients. To date, rTMS protocols used in stroke patients are relatively unified. However, as the pathophysiology of stroke is diverse and individual functional deficits are distinctive, more precise application of rTMS is warranted. Therefore, the objective of this study was to determine the effects of personalized protocols of rTMS therapy based on the functional reserve of each stroke patient in subacute phase.

**Methods:**

This study will recruit 120 patients with stroke in subacute phase suffering from the upper extremity motor impairment, from five different hospitals in Korea. The participants will be allocated into three different study conditions based on the functional reserve of each participant, measured by the results of TMS-induced motor evoked potentials (MEPs), and brain MRI with diffusion tensor imaging (DTI) evaluations. The participants of the intervention-group in the three study conditions will receive different protocols of rTMS intervention, a total of 10 sessions for 2 weeks: high-frequency rTMS on ipsilesional primary motor cortex (M1), high-frequency rTMS on ipsilesional ventral premotor cortex, and high-frequency rTMS on contralesional M1. The participants of the control-group in all three study conditions will receive the same rTMS protocol: low-frequency rTMS on contralesional M1. For outcome measures, the following assessments will be performed at baseline (T0), during-intervention (T1), post-intervention (T2), and follow-up (T3) periods: Fugl-Meyer Assessment (FMA), Box-and-block test, Action Research Arm Test, Jebsen-Taylor hand function test, hand grip strength, Functional Ambulatory Category, fractional anisotropy measured by the DTI, and brain network connectivity obtained from MRI. The primary outcome will be the difference of upper limb function, as measured by FMA from T0 to T2. The secondary outcomes will be the differences of other assessments.

**Discussion:**

This study will determine the effects of applying different protocols of rTMS therapy based on the functional reserve of each patient. In addition, this methodology may prove to be more efficient than conventional rTMS protocols. Therefore, effective personalized application of rTMS to stroke patients can be achieved based on their severity, predicted mechanism of motor recovery, or functional reserves.

**Clinical trial registration:**

https://clinicaltrials.gov/, identifier NCT06270238.

## 1 Introduction

### 1.1 Background and rationale

Stroke is still worldwide leading cause of disability, and the global burden has been increasing (1, 2). Impairment of upper limb motor function stands out as the foremost and prevalent sequelae of stroke, significantly impacting stroke patients’ independence in activities of daily living (3, 4). Therefore, clinicians are employing various therapies aimed at improving outcomes related to motor function, including conventional rehabilitative physical and occupational therapies.

Transcranial magnetic stimulation (TMS) generates magnetic field to induce electric currents in brain, using a magnetic coil. These currents primarily stimulate axons of the neural circuits and enable to assess motor cortex function or corticospinal connectivity (5). By recording motor evoked potentials (MEPs) in distal muscles following TMS, disease-related changes in corticospinal output can be assessed. Therefore, TMS has been proven useful as a predictor of motor recovery in stroke patients using measures such as motor threshold, amplitude and latency of MEP, cortical silent period, or central motor conduction time (5, 6). Since the early 2000s, the use of repetitive transcranial magnetic stimulation (rTMS) has emerged and is now widely utilized to enhance upper limb function in stroke patients, due to its feasibility, non-invasive nature and painless application (7–10). The underlying patho-mechanisms in applying rTMS therapy was mainly based on the neuroplasticity and the interhemispheric competition model theory (11–14). Applying rTMS to human cortex has been proven to modulate cortical excitability, leading to recovery or reorganization of the functional connectivity (5, 15). The neuroplasticity, in the context of stroke, is thought as the brain’s capacity to modulate its activity in response to stimuli, thereby compensating for damages resulting from stroke (16). rTMS therapy targets this plasticity by either inhibiting or exciting neural activity to induce or restore the desired plasticity in the brain (17, 18). In stroke patients in the acute phase, it is known that along with functional loss in the ipsilesional hemisphere, there are alterations in the interaction between the ipsilesional hemisphere and the contralesional hemisphere via the corpus callosum (19). It is thought that in regions remote from the brain lesion, there may be changes in neuroanatomy and cortical activity in both cerebral hemispheres (20). As a result, bilateral activation of both primary motor cortices is observed during movement in post-stroke patients, resulting in poor motor function compared to healthy people (21). Based on these theories, in stroke patients, many previous studies have demonstrated the effect of inhibitory low-frequency rTMS or continuous theta burst stimulation (cTBS) applied at contralesional primary motor cortex (M1) and facilitatory high-frequency rTMS or intermittent theta burst stimulation (iTBS) applied at ipsilesional M1 in enhancing upper limb function (22, 23).

The currently well-known conventional rTMS protocols for stroke patients involve applying inhibitory rTMS at the contralesional M1 or facilitatory rTMS at the ipsilesional M1. However, some studies demonstrated that these conventional rTMS protocols showed no significant effects when applied to severe hemiplegic stroke patients (24, 25). These results may imply that cortical activity or neural plasticity of individual stroke patients is not unified. Also, in cases where ipsilesional motor pathways are severely damaged, stimulating M1 may not be the optimized therapy for enhancing upper limb motor recovery. In fact, some previous studies have investigated the ipsilesional premotor cortex (PM) or supplementary motor area (SMA), and contralesional PM may replace the function of the damaged ipsilesional M1, although no consensus has been reached yet (26–29). Schulz et al. demonstrated a significant interaction between the corticospinal tract (CST) and corticocortical connections, implying that the ipsilesional ventral PM plays a role in patients with significant damage in CST (30). Sankarasubramanian et al. (31) also reported that the contralesional dorsal PM may support recovery in patients who have experienced extensive damage to ipsilesional motor pathways. In addition, Di Pino et al. (32) suggested a bimodal balance recovery model over the interhemispheric competition model. They suggested that interhemispheric balancing should be considered along with the functional reserve spared by every patient in the recovery model, not in isolation. Given the diverse underlying pathophysiology and recovery processes within stroke, this suggestion appeared reasonable. In 2018, Harvey et al. (33) demonstrated that applying inhibitory rTMS at ipsilesional M1 did not show effectiveness. Following the release of this trial, increasing inquiries have emerged regarding the rationality of applying conventional rTMS protocols based on the interhemispheric competition model. Ultimately, it is believed that employing a conventional rTMS approach, which applies the same protocol to everyone without considering individual characteristics, has limitations. Therefore, a consideration of individual functional reserve will be necessary for the implementation of rTMS tailored to each individual.

Besides the stimulating target of rTMS, another important consideration when applying rTMS therapy is the accuracy of the stimulation. The conventional rTMS treatment approach has historically positioned the area of maximal magnitude of the electric field induced by TMS along the central axis of the stimulation coil. Stimulation was conducted by aligning the coil to ensure that the central axis of the coil passed through the stimulation area. Additionally, determining the stimulation area was achieved by identifying the location that elicited the largest transcranial magnetic stimulation-induced motor-evoked potentials (TMS-induced MEPs), requiring numerous attempts of TMS to accurately ascertain the stimulation site (34). In addition, protocols based on anatomical landmarks or the 10–20 system have been used to stimulate non-motor areas where TMS-induced MEPs are not measured, which may increase the imprecision of rTMS targeting (35). Recently, a neuronavigation system is considered a viable method for obtaining accurate stimulation targets. However, a critical limitation of employing the neuronavigation is its expense, making it difficult to utilize in general environments (36). Recent advancements in neuroimaging techniques have enabled the development of computational brain modeling and electric field simulation techniques based on brain images such as magnetic resonance imaging (MRI) obtained from patients, which can address the limitations of conventional rTMS targeting methods (37, 38). Specifically, through the prediction and analysis of electric fields reflecting the unique anatomical information of the patient’s brain based on MRI, it has been revealed that the area of maximum magnitude of the electric field does not necessarily align with the central axis of the coil due to variations in brain structure (39, 40). Moreover, simulations have shown that when stimulating areas are targeted to achieve maximum field strength, actual TMS-induced MEPs are increased (41). Therefore, it is imperative to utilize electric field simulations and optimization processes based on brain imaging obtained from patients to determine the position and orientation of the TMS coil that will generate the optimal stimulation for the given target stimulation area. This approach should be applied to rTMS therapy to ensure its effectiveness.

Therefore, the aim of this study is to demonstrate the efficacy of rTMS protocols based on the functional reserve of individual stroke patients, including exploring the accurate stimulating target. We anticipate that our study protocols will demonstrate superiority over the conventional inhibitory rTMS protocol applied to the contralesional M1. Additionally, by utilizing the MRI of each individual patient, we aim to achieve accurate stimulation targets without relying on the neuronavigation, thereby offering convenience and cost-effectiveness for broader use. In addition, we would like to explore the mechanisms of personalized rTMS by performing serial resting-state functional MRI (rs-fMRI) and diffusion tensor imaging (DTI).

## 2 Methods and analysis

### 2.1 Study setting

This is a prospective, single-blind with blind observer, parallel-group design, multi-center, randomized controlled clinical trial. This study will recruit 120 patients with stroke in the subacute phase who are suffering from the upper extremity motor impairment, from five different hospitals in Korea. Participating hospitals are Samsung Medical Center, Seoul; Seoul National University Hospital, Seoul; Bucheon St. Mary’s Hospital, The Catholic University of Korea, Seoul; St. Vincent’s Hospital, The Catholic University of Korea, Seoul; Yongin Severance Hospital, Yongin.

**Figure 1:**
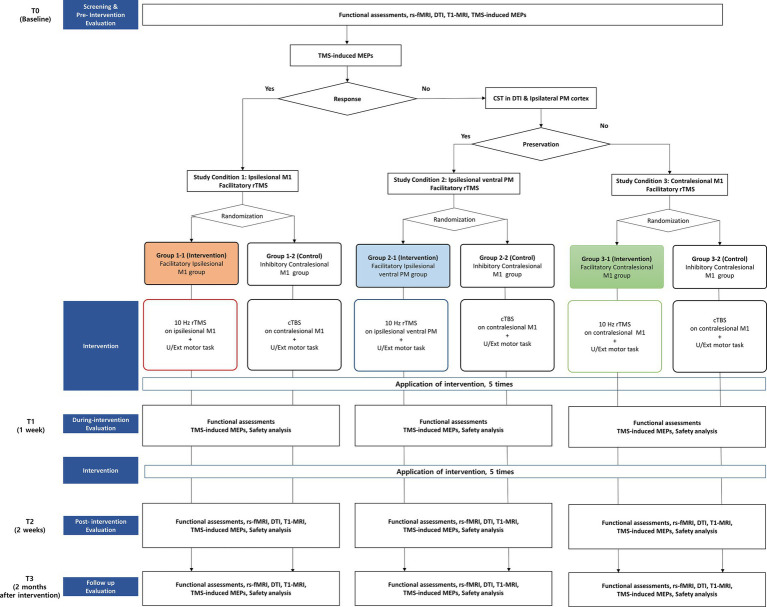
Design and flowchart of the study.

The participants will be allocated into three different study conditions according to their functional reserve, as follows: Study condition (1) participants with preserved ipsilesional CST, confirmed by response of TMS-induced MEPs, Study condition (2) participants with no response of TMS-induced MEPs, but with preserved ipsilesional PM cortex and ipsilesional CST confirmed by DTI, and Study condition (3) participants with no preservation of ipsilesional CST. After the allocation, participants will be randomly assigned to the intervention-group or control-group of each study condition through randomization. The participants of the intervention-group in the three study conditions will receive different protocols of rTMS intervention: Study condition (1) high-frequency rTMS on ipsilesional M1, Study condition (2) high-frequency rTMS on ipsilesional ventral PM, and Study condition (3) high-frequency rTMS on contralesional M1. The participants of the control-group in all three study conditions will receive the same rTMS protocol: low-frequency rTMS on contralesional M1. A schematic diagram is shown in Figure 1.

Evaluations to assess the functional reserve and motor function will be conducted as follows: (1) at baseline (T0), (2) after 1 week, following 5 sessions of rTMS intervention [during-intervention (T1)], (3) at the end of the rTMS intervention [post-intervention (T2)], and (4) 2 months after the end of the intervention [follow-up (T3)]. The specific timeline of participants is shown in Table 1.

**Table 1:** Timeline of enrolment, interventions, and assessments of this study.

	Enrolment	Baseline	Intervention (1 ~ 5)	During-intervention	Intervention (6 ~ 10)	Post-intervention	Follow-up
*Timepoint*		*T0*		*T1*		*T2*	*T3*
Informed consent	O						
Eligibility screen	O						
Allocation		O					
Assessments							
rs-fMRI		O				O	O
DTI		O				O	O
T1-MRI		O				O	O
TMS-induced MEPs		O		O		O	O
FMA		O		O		O	O
Box and block test		O		O		O	O
ARAT		O		O		O	O
Jebsen-Taylor hand function test		O		O		O	O
Hand grip strength test		O		O		O	O
FAC		O		O		O	O
Adverse events			O	O	O	O	O
Application of intervention							
Intervention			O		O		

rs-fMRI, resting-state functional MRI; T1-MRI, T1-weighted structural images of brain MRI; TMS-induced MEPs, transcranial magnetic stimulation induced motor evoked potentials; FMA, Fugl-Meyer Assessment; ARAT, Action Research Arm Test; FAC, Functional Ambulation Category.

### 2.2 Eligibility criteria

The inclusion criteria for this study are as follows: (1) hemiplegic stroke (ischemic or hemorrhagic stroke with corresponding lesion determined by MRI or computed tomography scan) patients in the subacute phase (7 days to 3 months from the onset) who are currently hospitalized, (2) Fugl-Meyer Assessment (FMA) score of the upper extremity ≤42, (3) adequate language and cognitive function to perform at least a 1-step obey-command, (4) pre-stroke functional level of modified Rankin Scale (mRS) ≤1, (5) aged ≥19 years old, and (6) patients willing to sign the informed consent. The exclusion criteria are as follows: (1) patients with transient ischemic attack, defined as a rapid-onset focal neurological deficit lasting less than 24 h (42) (2) those with contraindications to rTMS, such as epilepsy, implanted metal objects in the head, or a history of craniotomy, (3) those with progressive of hemodynamically unstable medical conditions, (4) those with coexisting neurological conditions, such as spinal cord injury or Parkinson’s disease, (5) those with major psychiatric disorders, such as major depression, schizophrenia, or dementia, (6) those having contraindications to conduct an MRI study, (7) those who are pregnant or lactating, and (8) patients who have refused to participate in this study.

### 2.3 Allocation

The 120 eligible participants will be allocated into three study conditions based on the functional reserve of each participant, measured by the results of the brain MRI, TMS-induced MEPs, and DTI evaluations. Each condition will consist of 40 patients and they will be randomly allocated into the intervention-group and control-group in a 1:1 ratio. The allocation will be performed by the one researcher of each participating hospital, who will be responsible for the randomization, will not have contact with the participant, and will not be involved in data collection or analysis. The randomization will be done by using a randomization table generated by the www.randomization.com. The randomization sequence will be concealed and only the research principal investigator (PI) will have access authority.

### 2.4 Blinding

The participants and assessors will be blinded, not be aware of the group allocation. Statistical analysis will also be conducted by data analysts without awareness of the group allocation. Only clinicians applying rTMS intervention will not be blinded, as they will apply rTMS over different stimulation sites based on the protocols. Blinding will be continued until the end of the study, including data analysis.

### 2.5 Intervention

The rTMS intervention will utilize either the Magstim Rapid^2^ (Magstim Co. Ltd., United Kingdom), or the MagPro X100 (MagVenture, based in Lucerne Marken, Denmark), employing the 70-mm figure-of-eight coil. The intervention will be applied as high-frequency protocols to the participants in the intervention-group in all three study conditions: 20 sessions of 10-Hz rTMS, 50 pulses per session with a 25-s interval between sessions, totaling 1,000 pulses (43). The difference among the three study conditions will be the targeted stimulation sites based on the functional reserve and stimulating intensity, as follows: (1) ipsilesional M1 and intensity set at 90% of the resting motor threshold (rMT) measured at contralateral first dorsal interosseous muscle (FDI) following stimulation of ipsilesional M1 for Group-1, (2) ipsilesional ventral PM and intensity set at 90% of the rMT measured at contralateral FDI following stimulation of contralesional M1 for Group-2, (3) contralesional M1 and intensity set at 90% of the rMT measured at contralateral FDI following stimulation of contralesional M1 for Group-3. The control-group of all three study conditions will be applied the same cTBS protocol for rTMS on contralesional M1, as follows: TMS pulses will be delivered as a 3-pulse burst at 50 Hz applied at 5 Hz for 40 s, with a stimulating intensity set at 70% of the rMT measured at contralateral FDI following stimulation of contralesional M1, totaling 600 pulses (44, 45). All participants will receive rTMS intervention once a day, 5 days per week, for 2 weeks, totaling 10 sessions of rTMS intervention.

The selection of the target stimulation site, specifically the ventral PM, will be manually identified using anatomical landmarks by an expert in neuroanatomy. Following the identification of the ventral PM, the Neurophet tES LAB software (NEUROPHET Inc., Seoul, Republic of Korea) will be employed. This software processes each participant’s T1-weighted brain images, which are acquired during the pre-intervention evaluation. The software then reconstructs these images into a three-dimensional model of the brain. Based on this model, the software provides guidance for the precise placement of the stimulation coil on the skin. The stimulating target of M1 will be identified using TMS-induced MEPs, where the maximum peak-to-peak amplitude in the contralateral FDI muscle is achieved (45, 46).

In addition, all participants will receive inpatient conventional rehabilitation therapy, consisting of occupational and physical therapy for 30 min each, twice daily, for 2 weeks, as well as the routine pharmacotherapy based on the guidelines for management of stroke patients (47–49).

During the intervention, participants are allowed to withdraw based on the following criteria: (1) those willing to withdraw, (2) loss to follow-up, (3) occurrence of adverse events, following withdrawal requests from participants, (4) other reasons deemed unsuitable for the progress of the study by the researchers.

## 3 Data collection

### 3.1 Brain imaging and cortical excitability

Brain imaging data, comprising rs-fMRI, DTI, and T1-weighted structural images, will be obtained using 3-T scanners (Philips Ingenia CX, Philips Elition, Siemens Magnetom Trio, Siemens Magnetom Vida). The rs-fMRI will be employed to extract brain networks through functional connectivity analysis. Alterations in brain network properties resulting from the intervention will be investigated by analyzing connectivity strength, employing graph theory, and conducting comprehensive assessments of both global and local networks, as well as intra- and inter-hemispheric networks (50). During the resting-state scan, participants will be directed to close their eyes and maintain stillness. Each session will involve the collection of 180 whole-brain images, utilizing the following metrics: 75 axial slices, slice thickness = 2 mm, no gap, matrix size = 112 × 112 or 124 × 124, and repetition time = 2000 ms. DTI will be utilized to extract the integrity of major neural pathways and structural networks through fiber tractography (51). It will also be used to investigate changes in the characteristics of integrity and networks resulting from the intervention (52). Each session will acquire more than 30 diffusion-weighted images with b = 1,000 s/mm2, ensuring a minimum of 75 axial slices, slice thickness = 2 mm, no gap, and matrix size = 112 × 112 or 128 × 128. Fractional anisotropy values (FA) of posterior limb of internal capsule (PLIC), and reconstructed corticospinal and corticobulbar tract will be obtained (53, 54). T1-weighted structural images will be used to ascertain the individual target positions of the ventral PM. These images will be acquired with a resolution and slice thickness of 1 mm or less to accurately guide the position of the TMS coil.

The cortical excitability of each participant will be measured, using the TMS-induced MEPs. TMS-induced MEPs will be assessed by single magnetic stimulations at 120% of the rMT over the M1 using a 70-mm figure-of-eight coil. During the experiments, participants will sit comfortably in an armchair with their eyes open. A Synergy electromyography/evoked potentials system (Medelec Co. Ltd., Kingswood, Bristol, United Kingdom) will be used to record and monitor the activity of the contralateral FDI muscle following stimulating over the M1 using single-pulse TMS. TMS will be applied using a BiStim^2^ stimulator (Magstim Co. Ltd., Spring Gardens, Whitland, Carmarthenshire, Wales, United Kingdom) equipped with a 70-mm figure-of-eight coil. The coil would be held tangentially to the scalp, with the handle pointing backward and laterally at 45° from the mid-sagittal line. Using TMS, the optimum position (“the hot spot”) will be defined as the site where TMS-induced MEPs of maximum peak-to-peak amplitude in the contralateral FDI muscle. We will define the rMT as stimulus percentage of maximal stimulator output (MSO) that elicits a minimum peak-to-peak amplitude of MEP over 50uV in at least 5 out of 10 trials (5). This rMT data will be used for determining the intensity of the rTMS intervention in this study. Including the aforementioned rMT, amplitude and latency of MEP will be recorded as TMS-induced MEPs data. To measure the amplitude and latency, the intensity of the TMS stimulation will be set at 120% of the measured rMT. The stimulation will be repeated 10 times, with intervals of 5 s or more. The average peak-to-peak amplitude and latency of the top 5 responses will be measured and recorded (55). The latency will be the time between the onset of the TMS stimuli and the onset action potential (56). In addition, medications known to have potential effects on MT or MEP and the risk of seizures associated with rTMS will also be documented (15, 57).

### 3.2 Functional assessment

Functional assessments will be conducted at T0, T1, T2, and T3 periods in each participating hospital. To maintain the data quality and inter-rater reliability, the assessors will be trained before the start of the study and uniform manuals will be shared with the assessors and research investigators. In this study, all functional assessment tools selected are widely used for stroke patients and have been frequently employed in previous studies using rTMS (22, 23). Additionally, their reliability and validity have been proven. For motor function, FMA will be used. FMA measures the movement, reflexes, coordination, and speed of limbs (58, 59). The total, upper extremity (UL), and lower extremity (LL) score of FMA will be assessed separately. For hand function assessments, the following tests will be used: the Box and Block Test, reflecting clinical manual dexterity (60, 61); the Action Research Arm Test (ARAT), measuring gross motor skills, grasp., grip, and pinch (62); the Jebsen-Taylor Hand Function Test, assessing the fine motor function of the hand used in daily activities (63, 64); and the Hand Grip Strength Test (65). The Functional Ambulation Category (FAC) will be used to assess ambulatory function, categorizing patients by their level of dependence in walking (66).

### 3.3 Outcomes

The primary outcome of this study is difference of FMA-UL from baseline (T0) to post-intervention (T2). The secondary outcomes of this study are as follows:

1. Differences of FMA-total, FMA-UL, FMA-LL, Box and block test, FAC, ARAT, Jebsen-Taylor hand function test, hand grip strength test from baseline (T0) to during-intervention (T1).
2. Differences of FMA-total, FMA-LL, Box and block test, FAC, ARAT, Jebsen-Taylor hand function test, and hand grip strength test, FA of PLIC, FA of reconstructed corticospinal and corticobulbar tract, global and local connectivity obtained from rs-fMRI from baseline (T0) to post-intervention (T2).
3. Differences of FMA-total, FMA-UL, FMA-LL, Box and block test, FAC, ARAT, Jebsen-Taylor hand function test, and hand grip strength test, FA of PLIC, FA of reconstructed corticospinal and corticobulbar tract, global and local connectivity obtained from rs-fMRI from baseline (T0) to follow-up (T3).

### 3.4 Sample size, recruitment

Based on the primary outcome of this study and previous literature, we have established the study’s power (1-β) at 80% and a significance level (α) of 5%. The clinically significant effect size (δ) has been designated as 7.25, with an expected standard deviation (σ) of 7.70 (67, 68). Sample size calculation was conducted using Lehr’s formula, with an expected follow-up rate of 90% (68, 69). Finally, the calculated sample size was 120, with each subgroup consisting of 40 participants.

Recruitment of the study participants will be conducted by each participating hospital.

Every research investigator will recruit participants from each hospital who are eligible to participate in the study. Participants will be informed about the current treatment guidelines regarding upper extremity dysfunction in stroke patients, as well as the potential and adverse effects of this study.

### 3.5 Statistical methods

Demographic and clinical characteristics were reported in terms of frequencies and percentages for categorical variables, while means and standard deviations (SD) were utilized for numerical variables. To compare baseline characteristics of the intervention and control group, an independent t-test and the Wilcoxon Signed-rank test will be used for normally distributed and non-normally distributed variables, respectively. The normality of each variable will be examined using the Shapiro–Wilk test.

All participants undergoing intervention in this study will be included in the intention-to-treat (ITT) set. Safety analyses will be conducted based on the ITT set. Participants who completed all of the evaluation regarding the study protocol will be classified as per-protocol (PP) set. The efficacy analyses will be conducted based on the ITT set. For missing values, data will be analyzed using the Last Observation Carried Forward (LOCF) method. Efficacy analyses well be conducted on the primary and secondary outcomes. For outcome variables, the normality will be examined using the Shapiro–Wilk test. If variables demonstrate normal distribution, a repeated measures analysis of variance (RM-ANOVA) will be used to evaluate the effect of time and groups, including the interaction. If non-normality is found, the Friedman test will be used to determine the differences between the groups. During the analyses, baseline characteristics will be used as covariates for adjustment. In addition, independent t-test or Wilcoxon Signed-rank test will be used to compare parameters between the intervention group and the control group in each condition. Statistical significance will be set at a *p*-value <0.05 for all the analyses.

### 3.6 Data management and monitoring

All data will be collected using standardized electronic Case Report Form (eCRF) and study participants will be identified only by a research-specific serial number. All personal information and collected data of participants will be maintained in confidentiality under the responsibility of research PI of each participating hospital. All personal information and collected data of participants will be maintained in confidentiality under the responsibility of the research PI of each participating hospital. They will be stored in password-protected files and kept in a locked facility. Routine supervision of the data will be conducted by one of researchers in each participating hospital, independent of other research investigators. Data analysis will be conducted by data analysts, independent of other research investigators. PIs will meet every month to review the implementation of this study. There are no conflicts of interest among all participating researchers.

### 3.7 Adverse events

Adverse events expected in this study include discomfort, dizziness, nausea, headache, hearing disturbance, pruritus, allergic reaction, or localized pain, as described in previous studies. The most serious expected side effect if a seizure; however, the occurrence of seizure is rarely reported (14, 22). All adverse events will be monitored. All adverse events that occurred will be reported to research principal investigators, ethics committee of each participating hospital, and the Ministry of Food and Drug Safety of the Republic of Korea within 7 days.

## 4 Discussion

The objective of this study was to determine the effects of protocols of rTMS therapy based on the functional reserve of each hemiplegic stroke patient in subacute phase, compared to conventional low-frequency rTMS therapy on contralesional M1. To the best of our knowledge, this is the first study willing to determine the effects of applying different protocols of rTMS therapy based on the functional reserve of each patient.

From this study, we anticipate several advantages distinct from those of previous studies. Firstly, this approach could be more effective compared to unified conventional rTMS protocols applied to stroke patients regardless of their severity. The main purpose of this study protocol is to validate a strategy based on a predicted mechanism of motor recovery, defined as functional reserves, that can overcome the limitations of conventional rTMS methods. Although the concept of functional reserves in stroke patients has been proposed, there is a lack of research on the application of stroke rehabilitation strategies using functional reserves (16, 70, 71). If successful, this study is expected to serve as a basis for the application of new rehabilitation strategies utilizing functional reserves, in addition to suggesting a new personalized approach to the application of rTMS in stroke patients.

Secondly, instead of relying on the expensive neuronavigation system, we intend to select accurate stimulation targets based on the MRI of each individual stroke patient. By reducing the economic expense while maintaining accuracy in rTMS therapy, this protocol method could be more conveniently utilized in various situations, facilitating further treatment and research. In particular, it is expected to provide an effective strategy that can be applied to rTMS targeting methods where it is difficult to determine the stimulation location with TMS-induced MEPs, such as the PM, SMA, dorsolateral prefrontal cortex, and cerebellum.

Lastly, by combining the analysis of serial MRI and DTI evaluations with the results of the personalized rTMS protocols in this study, we expect to approach the underlying mechanisms of rTMS therapy in enhancing upper limb motor recovery in stroke patients. The practical validation of functional reserves and the results on neurophysiological mechanisms of personalized rTMS are expected to serve as a basis for future studies on the improvement of upper limb function in stroke patients.

## Ethics and dissemination

The PI of the Samsung Medical Center will be the Chief Investigator (CI) of this study. The CI will inform the other PIs of each participating hospital regarding important protocol amendments. The CI will report these amendments to the Ministry of Food and Drug Safety of the Republic of Korea. The PI of each participating hospital will report these amendments to their respective ethics committee and research teams.

Prior to inclusion of participants, all participating hospitals obtained institutional review board (IRB) approval for this study (Samsung Medical Center, 2023–11-164; Seoul National University Hospital, 2,312–167-1498; Bucheon St. Mary’s Hospital and St. Vincent’s Hospital, The Catholic University of Korea, XC24DND30004; Yongin Severance Hospital, 9–2024-0013). If any protocol modifications are needed, further approval from the IRB will be obtained from all participating hospitals. Informed consent will be obtained from all participants prior to their inclusion in this study by research investigators. In addition, this study has been registered in the clinicaltrials.gov (NCT06270238). The results of this study are expected to be published within 2 years of its completion.

## Author contributions

HL: Investigation, Writing – original draft, Methodology. DHK: Project administration, Conceptualization, Writing – review & editing, Supervision. HS: Project administration, Conceptualization, Writing – review & editing, Supervision. SI: Project administration, Writing – review & editing, Supervision, Conceptualization. YY: Writing – review & editing, Investigation, Methodology. NK: Project administration, Writing – review & editing, Conceptualization, Supervision. JL: Project administration, Writing – review & editing, Supervision, Conceptualization. DK: Project administration, Writing – review & editing, Supervision, Conceptualization. H-YP: Writing – review & editing, Investigation, Methodology. M-JY: Writing – review & editing, Methodology, Investigation. YK: Writing – review & editing, Methodology, Investigation. HK: Writing – review & editing, Methodology, Investigation. WC: Writing – review & editing, Supervision, Project administration, Funding acquisition, Conceptualization.
